# Corrigendum to: Does small equal predatory? Analysis of publication charges and transparency of editorial policies in Croatian open access journals

**DOI:** 10.11613/BM.2017.031202

**Published:** 2017-10-15

**Authors:** Jadranka Stojanovski, Ana Marušić

**Affiliations:** 1 University of Zadar, Zadar; 2 Ruđer Bošković Institute, Zagreb, Croatia; 3 University of Split School of Medicine, Split, Croatia

This is a correction for *Biochemia Medica* 2017;27 (*2*): 292-9. DOI: https://doi.org/10.11613/BM.2017.032

In our article „Does small equal predatory? Analysis of publication charges and transparency of editorial policies in Croatian open access journals“ published in 2017 June issue of the *Biochemia Medica*, we made an unintentional error regarding the number of Croatian journals receiving support from the Croatian Ministry of Science and Education (MSE). In our analysis of Croatian open-access journals in the Hrčak repository (444 journals), we presented the data on number of journals from Hrčak subsidized by the MSE in 2016 (175 journals).

Because of the inconsistencies in the identification of the journals, we miscategorized 2 out of 175 journals receiving the financial support from MSE as not being included in the Hrčak repository. The correct statement should be that all journals supported by the MSE were in the Hrčak repository. This correction in no other way influence the results and the message of our study.

For our analysis, we have used the list available at the MSE site (https://mzo.hr/sites/default/files/dokumenti/2017/02/znanstveni_casopisi_i_casopisi_za_popularizaciju_znanosti_odobreni_u-2016_godini.pdf) and the list of Hrčak journals, available at http://hrcak.srce.hr/stat.php. The MSE list includes the name of the publisher, journal title, scientific field, requested and approved financial support in 2016. The list does not include the International Standard Serial Number (ISSN), which is the unique identifier of a journal. The list at Hrčak includes the journal title, status in Hrčak, ISSN (online), ISSN (print), URL of the journal, coverage, number of articles for different criteria, date of journal inclusion, date of the publication of the last issue and date of the last Hrčak upload. This list does not include subtitles of the journal, which are a part of the bibliographic records and are visible at other Hrčak web-pages. As both lists have journal titles, we presumed that automated comparison of the journal titles would give the correct matching of the titles in the two lists. During our analysis, two journal titles were not matched because journal titles differed and we stated in the published article that two journals that received the financial support from the MSE were not included in the Hrčak repository. Since the financial support of the journals was not the focus of the study, we did not perform a check for each of the individual journals.

The first journal, present on the MSE list of funded journals under the title *Radovi Zavoda za hrvatsku povijest* (without ISSN and subtitle), carries the main title of *Radovi* in the Hrčak repository (ISSN Print 0353-295X, ISSN Online 1849-0344). The same journal title *Radovi* is also present in the catalogue of the National and University Library (NUL) and their ISSN office, and on the journal cover page itself. The subtitle in Hrčak is *Radovi Zavoda za hrvatsku povijest Filozofskoga fakulteta Sveučilišta u Zagrebu*. Scopus and Web of Science Core Collection (WoSCC) databases index the journal as *Radovi Zavoda za Hrvatsku Povijest* with the correct ISSNs. On its own website, the journal cites the title *Radovi Zavoda za hrvatsku povijest* but the issues themselves are listed under the title *RADOVI*.

The second journal, present on the MSE list of funded journals as *Hrvatski časopis za odgoj i obrazovanje* (without ISSN and without subtitle), carries the main title of the *Croatian Journal of Education* in the Hrčak repository (ISSN Print 1848-5189, ISSN Online 1848 -5197) and the subtitle *Hrvatski časopis za odgoj i obrazovanje*. In the NUL catalogue, the journal has the Croatian title *Hrvatski časopis za odgoj i obrazovanje*. WoSCC indexes it as the *CROATIAN JOURNAL OF EDUCATION-HRVATSKI CASOPIS ZA ODGOJ I OBRAZOVANJE* and Scopus indexes it as the *Croatian Journal of Education*, both with the correct ISSNs.

Inconsistent presentation of journal information in the online environment is not rare in the global research community and has been discussed by several studies (*1*). A lot of effort has been invested to raise the awareness of the problem with the variations of journal titles and subtitles in different catalogues, repositories, and databases among Croatian editors and publishers and the importance of the consistent and standardized usage of the journal title, ISSN, and other citation elements. The lack of accurate identifiers, including ISSN, for all titles and formats within different journal lists, platforms and websites can cause problems in the journal identification (*2*). We hope that all stakeholders involved, especially the MSE and the Hrčak repository will harmonize the information present in the public domain and ensure the unique identification of Croatian scientific journals.

## References

[1] Boissy B Romano Reynolds R Boyd M Humble PIE-J and What [is] ISO 8: National and International Efforts toward Improved Journal Presentation. Ser Libr. 2012;62:206–12. 10.1080/0361526X.2012.652933

[2] Glasser S Broken links and failed access. Libr Resour Tech Serv. 2012;56:14–23. 10.5860/lrts.56n1.14

